# Signs Analysis and Clinical Assessment: Phase-Contrast Computed Tomography of Human Breast Tumours

**DOI:** 10.1371/journal.pone.0124143

**Published:** 2015-04-06

**Authors:** Wushuai Jian, Mingshu Wu, Hongli Shi, Liting Wang, Lu Zhang, Shuqian Luo

**Affiliations:** School of Biomedical Engineering, Capital Medical University, Beijing, China; Institute of Automation, Chinese Academy of Sciences, CHINA

## Abstract

**Purpose:**

To analyse the diagnostic signs present in slices of human breast tumour specimens using synchrotron radiation phase-contrast imaging computed tomography (PCI-CT) for the first time and assess the feasibility of this technique for clinical applications.

**Materials and Methods:**

The ethics committee of our university and relevant clinical hospital approved this prospective study, and written informed consent was obtained from all patients. PCI-CT of human breast tumour specimens with synchrotron radiation was performed at the Shanghai Synchrotron Radiation Facility (SSRF). A total of 14 specimens of early-stage carcinomas and 8 specimens of adenomas were enrolled. Based on raw data reconstruction, the diagnostic signs present in the slices were analysed and correlated with histopathology. We proposed a criterion for clinical diagnosis according to the evaluated signs and the Breast Imaging Reporting and Data System (BI-RADS) for reference. The criterion was then assessed by clinicians in a double-blind method. Finally, descriptive statistics were evaluated, depending on the assessment results.

**Results:**

The 14 carcinoma specimens and 8 adenoma specimens were diagnosed as malignant and benign tumours, respectively. The total coincidence rate was 100%.

**Conclusion:**

Our study results demonstrate that the X-ray diagnostic signs observed in the specimen slices and the criterion used for clinical diagnosis were accurate and reliable. The criterion based on signs analysis can be used to differentiate early-stage benign or malignant tumours. As a promising imaging method, PCI-CT can serve as a possible and feasible supplement to BI-RADS in the future.

## Introduction

Breast cancer is the most common cancer among women worldwide. Early diagnosis and treatment is particularly crucial to reducing the mortality rate [1–4]. The routine method for identifying breast lesions is digital mammography. The modalities for breast imaging have undergone substantial improvements over the past 10 years[5–7]. Nevertheless, due to limited spatial resolution, the current modalities for breast screening suffer from a higher false-positive rate and often require a second test and follow-up. Moreover, according to the relevant literature, dense breast generates a higher false-negative rate[8–11]. The sensitivity ranges from 60% to 90%, and the specificity ranges from 80% to 95% for different diagnostic modalities with respect to previous investigations [10–11]. Thus, the presently available modalities for breast screening cannot fulfil the current demands for increasing the detection rate and reducing the false-negative rate at an earlier stage of breast tumour, especially for malignant lesions. The primary imaging tool for breast screening over the next decade will likely be high-resolution, high-contrast, anatomical X-ray imaging [6].

Phase-contrast imaging is one of the most important emerging X-ray imaging techniques [12–17]. In principle, this imaging technique relies on the phase shift that X-rays undergo when passing through matter [18–21]. The introduction of phase-contrast methods in breast imaging has provided improved contrast and dose reduction and has allowed high resolution CT imaging, and these methods can be transferred to the clinical environment. Phase-contrast imaging computed tomography (PCI-CT) with synchrotron radiation is a revolutionary X-ray imaging technique [22–25].The X-rays emerging from the specimen at different angles propagatethrough free space until they reach the detector. When the source exhibits high coherence and the detector is placed at a proper distance behind the sample, one will observe a fringe pattern while the different components of the beam, after diffraction by the sample, interfere with one another upon their further propagation through space. The interference pattern contains useful phase information. For in-line phase-contrast imaging (IL-PCI), the detector is placed sufficiently far behind the sample that the wave front distortions generated by the sample produce interference fringes at the detector. At an appropriate object-image distance, these fringes yield edge enhancements in the image [26–28]. The X-ray ILPCI experiments were performed using the BL13W1 beamline of the Shanghai Synchrotron Radiation Facility (SSRF) [20]. Previous studies have reported improvements in image quality afforded by the use of IL-PCI mammography. However, to our knowledge, the investigators in previous studies have not discriminated signs in slices generated from PCI-CT reconstruction and prospectively summarisedcriteria for clinical diagnosis, similar to those provided by BI-RADS [29–31]. Therefore, our study emphasised the assessment of the diagnostic contribution of PCI-CT and summarises the characteristics of images for future prediction applications.

## Materials and Methods

### Specimen Collection and Preparation

The ethics committee of Capital Medical University granted approval for this study, and written informed consent was obtained from all patients. A total of 22 cases of human specimens in vitro were enrolled. The samples were fixed in 10% neutral-buffered formaldehyde solution. A total of 721 projection images of the sample were acquired when the samples was rotated over 180 degrees. Image acquisition was achieved using a CCD (Charge Coupled Device) camera (SSRF pixel size 9 μm; number of pixels 4000×2500). All formaldehyde-fixed specimens were from female patients. All specimens had undergone surgical resection and histological examination. Among the tissue specimens, there were 14 cases of invasive ductal carcinoma (IDC) and 8 cases of fibroadenomas. The size of the samples was approximately 10 mm×5 mm×2 mm, and the samples were kept as dry as possible prior to the start of the experiment. After collecting the projection images, the formaldehyde-fixed samples were dehydrated before being embedded in hot paraffin wax. After solidification, the paraffin blocks were cut into 9-μm sections using a standard microtome, and the sections were stained with haematoxylin and eosin using standard protocols. We obtained panoramic pictures of the pathological slices using a PRECICE 511 Digital Scanning System in the pathology department of the Capital Medical University-affiliated Beijing Friendship Hospital. The image viewer used was PRECICE iViewer 3.3.4, TMAP format, with a picture size of 435 MB-1.1 GB and a maximum magnification of 100×.

### Synchrotron Radiation Facility

**Fig 1:**
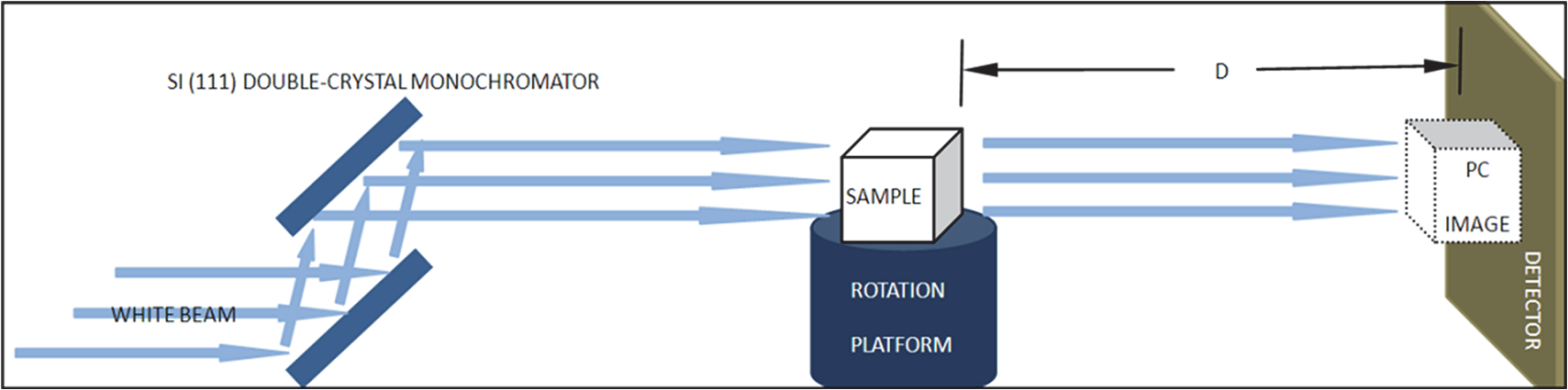
Schematic image of the experiment layout, which consisted of two monochromator crystals, one automatic rotation sample stage and one X-ray sensitive CCD detector. The incident white synchrotron X-ray beam was first monochromatised by two Si (111) prefect crystals. Subsequently, the highly parallel and monochromatic beam projected on the object was imaged. D denotes the distance between the object and the detector.

The synchrotron radiation PCI experiment for breast specimens was performed using X-ray imaging and the biomedical application beamline (BL13W1) of the Shanghai Synchrotron Radiation Facility (SSRF). The SSRF can perform in-line X-ray PCI and is the third-generation synchrotron radiation source in China [32–34]. Fig 1 shows a schematic image of the experimental layout. The device consists of two monochromator crystals, one automatic rotation sample stage, and one X-ray sensitive CCD detector. The incident white synchrotron X-ray beam was first monochromatised by two Si (111) prefect crystals. Subsequently, the highly parallel and monochromatic beam projected on the object was imaged.

The synchrotron radiation energy level ranged from 17 to 22 keV, according to the specimen’s thickness and density. The energy selection was evaluated using a phantom simulation prior to the specimen experiment. An exposure curve was used to adjust the energy to increase contrast of the breast parenchyma, stroma, tumour tissue, or normal glandular tissue. The distance between the sample and the CCD detector was 1.2 m. All specimens were kept as flat and smooth as possible, and the largest facet of the specimen was maintained parallel to the sample platform to facilitate the subsequent image reconstruction and pathological slicing.

### Image Processing and Tomography Reconstruction

**Fig 2:**
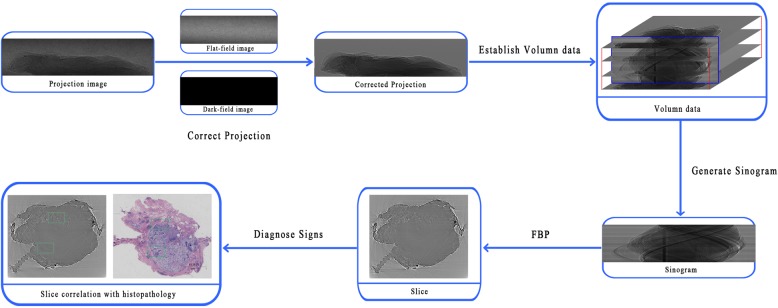
Flow path of data processing. The boxes represent data input, data processing, and data output, and the arrows denote the data flow direction. Slice is the purpose image for analysing and summarising the diagnostic signs.

X-ray PCI utilises phase shifts information as the imaging signal. At the BL13W1 beam line at SSRF, this technique was classified as propagation-based PCI. Because the phase information is embedded in the recorded images and cannot be accessed directly, further processing is required. The data-processing principle includes flat-field and dark-field correction, sinogram generation, and slice reconstruction. Initially, two-dimensional projection images were obtained. To obtain the final CT slices, image processing was required. The flat-field and dark-field images were initially used for correction. In the experiment, the monochromator may introduce background features into the illuminating beam. Moreover, if the beryllium windows and filters are not properly polished, artefacts will be generated. All of these factors affect the intensity of the projection image. To correct the intensity modulations, the formula for the corrected projection I was used: where I_p_, I_f_,and I_d_ represent the projection image, flat-field image, and dark-field image, respectively. The next step was to generate the sinogram. The sinogram represents the signal measured along a given detector row in the imaging plane for all CT scan angles. The method to generate the sinogram was as follows. First, an empty volume data was establish in the computer memory, the size of which corresponded to the projection size and numbers of CT projections. Second, the corrected projections were loaded in sequence. Third, we obtained the final sinogram after saving the row plane. Finally, the traditional algorithm FBP (filtered back-projection) was used in the tomographic reconstruction [35]. Fig 2 shows the flow path of data processing.

The 3D reconstruction step was performed using the Amira 5.2 software.Amira is a powerful, multifaceted 3D software platform for visualising, manipulating, and understanding biomedical data arising from all types of sources and modalities.

### Image Evaluation and Clinical Assessment

**Fig 3:**
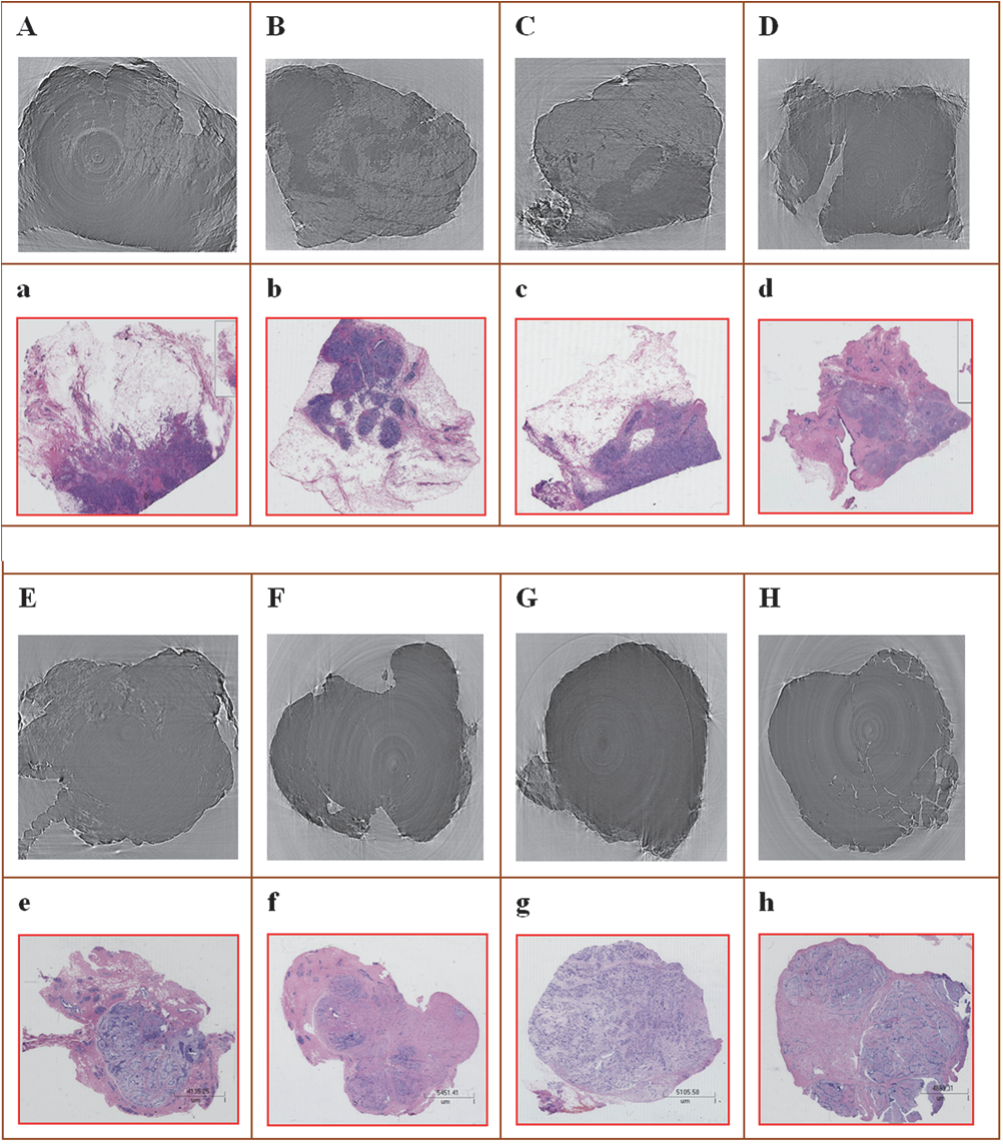
The correlation between the diagnoses determined from the PCI-CT slices and the histopathological results. Images A to D are slices of four cases of IDC. Images a tod are their corresponding histopathological images. Images E to H are slices of four cases of FA. Images e to hare their corresponding histopathological images.

In total, there were 22 specimens in our experiment. Hundreds of slices were reconstructed for each specimen. To assure objectivity during clinical assessment, two types of image datasets were arranged: an analytic dataset and a clinical dataset. We analysed the signs present in the analytic dataset slices and established a diagnostic criterion. Then, five radiologists judged the reliability of the criterion using the clinical dataset. The diagnostic signs present in the imaging slices were analysed in regards to the tissue pathology, anatomy, and medical imaging diagnosis and were correlated with the final pathological results. Finally, with reference to BI-RADS, we established a diagnostic criterion. To test its reliability, a double-blind examination was conducted. The clinical dataset in the aforementioned evaluation was used, but the order of the samples was rearranged, and the diagnostic results were unknown. In particular, for the same specimen, the slices in the clinical dataset differed from those in the analytic dataset. Five senior radiologists with at least 8 years of experienceassessed the diagnostic signs present in the imaging slices and arrived at a conclusion concerning whether the lesion was malignant or benign according to the criterion. Prior to evaluating the testing image dataset, the radiologists were trained using the analytic image dataset. The radiologists observed the slices carefully and assessed whether the samples exhibited diagnostic signs that completely or partially conformed to the criterion. The decisions were documented with a mark in the test table for forthcoming statistics. Fig 3 shows the excellent consistency between the slices and histological images.

**Fig 4:**
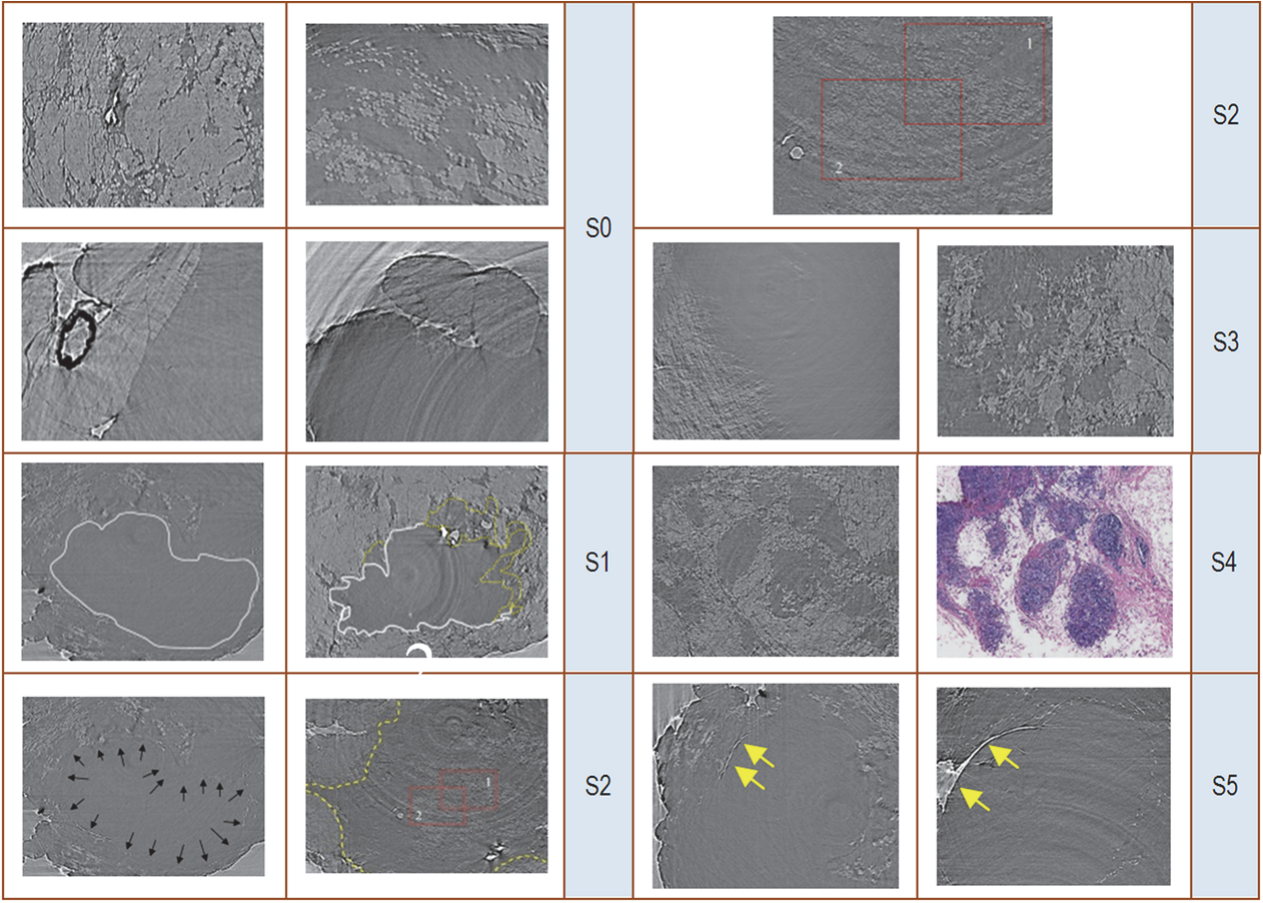
The established signs S0 to S5. S0, scattered or lump-like adipose tissue around the tumour. S1 (silhouette sign), irregular margins or spiculation of the tumour.S2, scattered adipose tissue within the tumour area.S3, peripheral distorted and disordered adipose tissue.S4 (finger pressure sign), finger-like impressions in the interface region between the tumour and surrounding structures. S5 (border sign), refers to a natural line that at least partially outlines the tumour contour.

We obtained 6 types of signs, ranging from sign S0 to sign S5, that were primarily determined according to the distribution of adipose tissue and the edge characteristics of the lesion.S0 refers to scattered or lump-like adipose tissue around a tumour. This feature was observed in healthy or hyperplastic breast. S1, termed the silhouette sign, refers to an irregular margin or spiculation of the tumour.S2 refers to scattered adipose tissue within the tumour area.S3 refers toperipheral distorted and disordered adipose tissue. S4, termed the finger pressure sign, refers to finger-like impressions in the interface region between the tumour and surrounding structures. S5, termed the border sign, refers to a natural line that at least partially outlines the tumour contour. Fig 4 illustrates the six signs, from sign S0 to sign S5.

In Table 1, the frequency of various signs observed in the analytic dataset is reported. According to Table 1, we acquired a relative scoring standard for the discrimination of malignant and benign tumours in terms of the importance and interpretation of signs. We categorised specimens with total scores greater than or equal to 9 as malignant, while samples with total scores lesser than or equal to 3 were categorised as benign lesions. If the total scores were between 3 and 6, the lesion could not be adequately classified and required further clinical examination. The above standards served as a diagnostic criterion for radiologists to assess the clinical dataset.

**Table 1:** Frequency Record of Signs in the Analytic Dataset.

**Signs S0-S5**	**Malignant Tumour(14 Cases)**	**Benign Tumour(8 Cases)**	**Scoring Standard**
S0	14	8	0
S1	14	0	3
S2	14	2	3
S3	14	0	3
S4	6	0	2
S5	0	6	-3

### Statistical Analyses

Descriptive statistics were applied. The sensitivity and specificity were simply evaluated.

## Results

**Fig 5:**
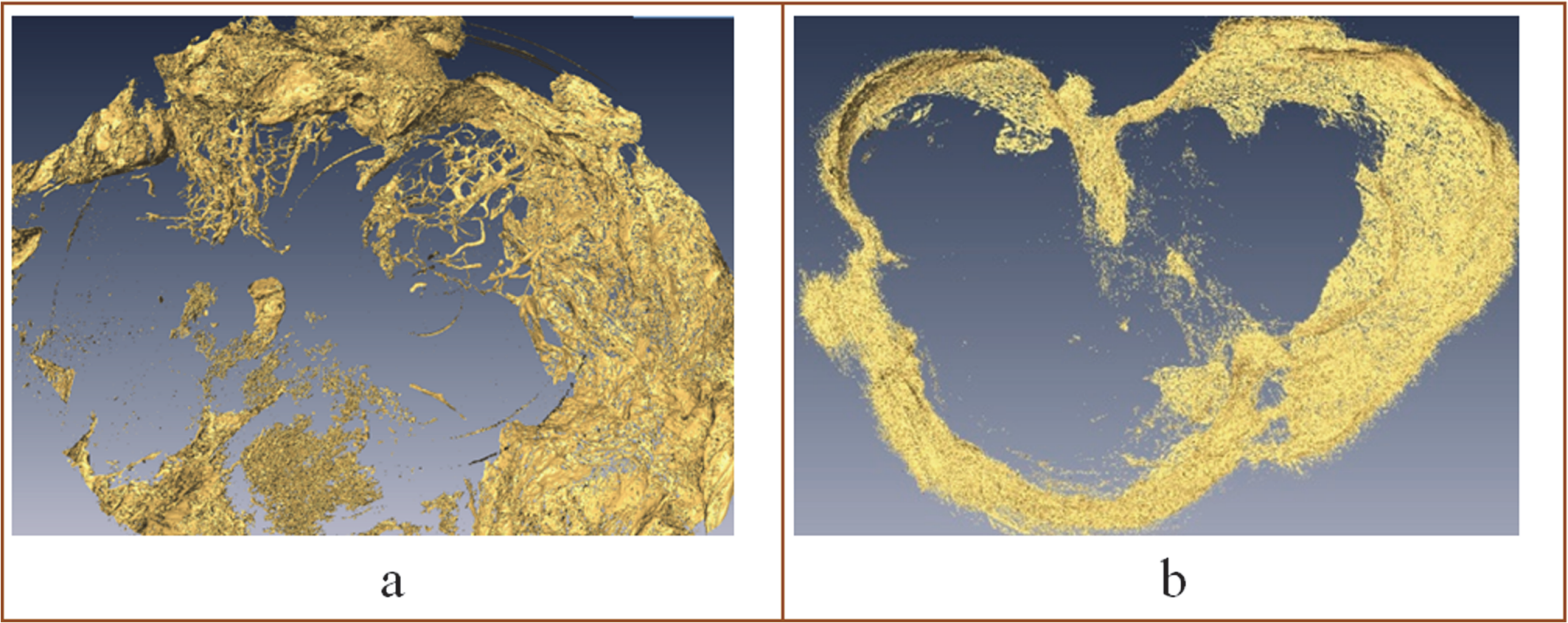
3D reconstruction images of the slices. (a) Reconstructed image of a malignant tumour. There are various distributions of adipose tissue surrounding the tumour, and scattered adipose tissue can be observed within the tumour area. (b) Reconstructed image of a benign tumour. The lesion has a regular margin, and there is no scattered adipose tissue within the tumour area.

**Fig 6:**
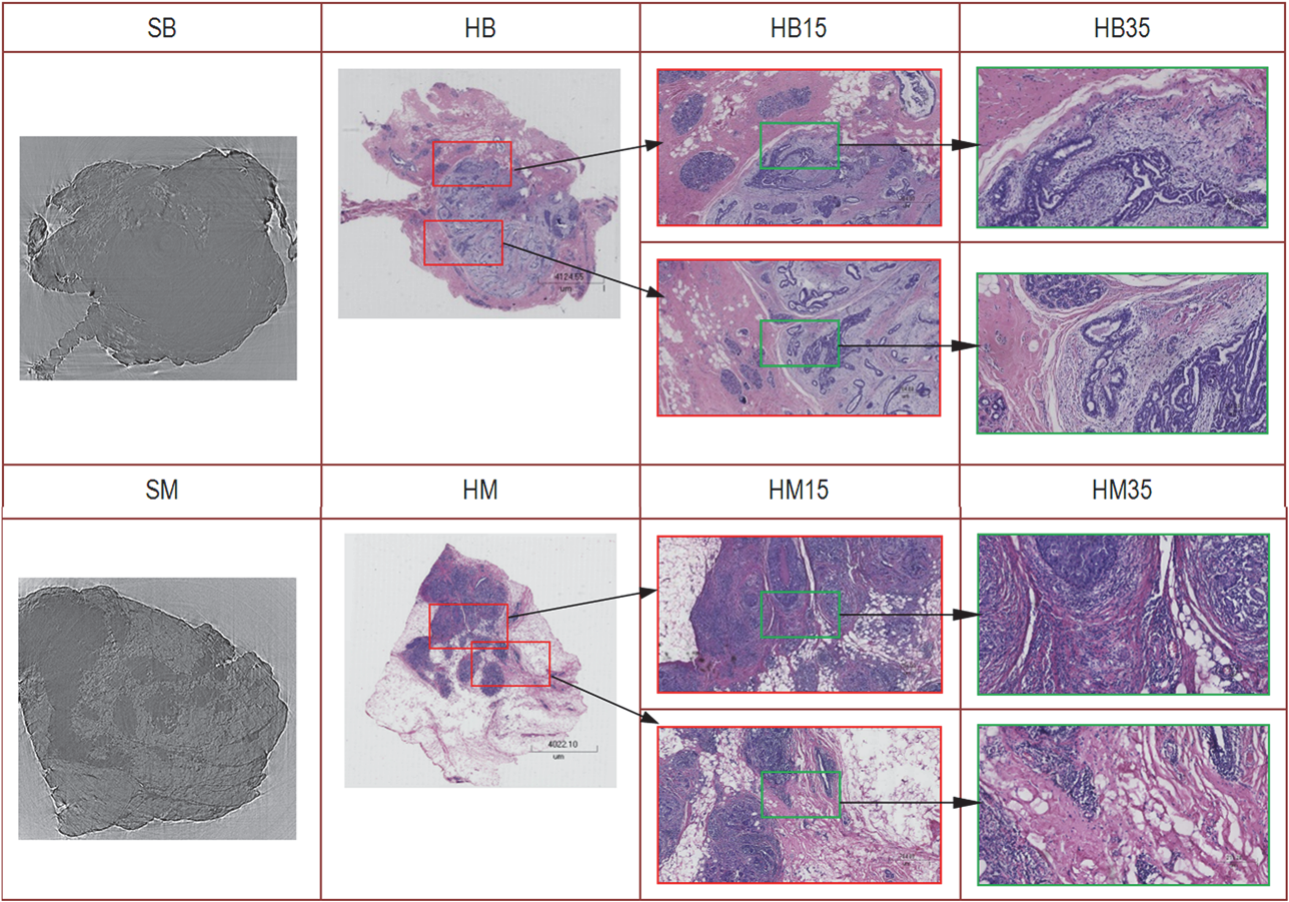
Correlation between the slice and histopathological results in detail. In the upper row, SB is a slice of a case of FA. HB is the corresponding histopathological image. HB15 and HB35 are detailed images of the histopathological results, at magnification 15×and magnification 35×, respectively. In the bottom row, SM is slice of a case of IDC. HM is the corresponding histopathological image. HM15 and HM35 are detailed images of the sample histopathology, at magnification 15×and magnification 35×, respectively.

The final diagnoses achieved with the reference standards were 8 benign findings and 14 malignancies. Table 2 shows the results for the clinical image dataset assessed by radiologists. In comparison to BI-RADS, CT with synchrotron radiation yielded 22 true-positive findings. Consequently, the sensitivity and specificity of mammography with synchrotron radiation computed tomography for our samples were both 100%. All five radiologists obtained correct results. There were no false-negative findings. No benign lesions were diagnosed as malignancies, and no malignancy was diagnosed as a benign lesion. In the assessments with signs S2 and S5, there were subtle differences in scoring. Among the eight benign tumours, there were two cases of S2 items and six cases of S5 items, with no discernable regularity to the recorded results. The possible causes for this inconsistency are analysed in the conclusion section. Fig 5 shows 3D reconstructed images of slices of malignant and benign tumours that were generated using the AMIRA software. Various distributions of adipose tissue surrounding the tumour and scattered adipose tissue within the tumour area can be clearly observed. Fig 6 shows details of the histopathological results correlated with those from the PCI-CT slices.

**Table 2:** Scoring Results for the Clinical Dataset Evaluated by Radiologists.

**No. Specimens**	**Scoring by Radiologists (Corresponding Items)**					**Histopathological Results**
	**A**	**B**	**C**	**D**	**E**	
**1**	9(S0123)	9(S0123)	11(S01234)	11(S01234)	9(S0123)	IDC*
**2**	0(S025)	-3(S05)	-3(S05)	-3(S05)	0(S035)	FA*
**3**	9(S0123)	9(S0123)	9(S0123)	9(S0123)	9(S0123)	IDC
**4**	9(S0123)	9(S0123)	9(S0123)	9(S0123)	11(S01234)	IDC
**5**	9(S0123)	9(S0123)	9(S0123)	9(S0123)	9(S0123)	IDC
**6**	11(S01234)	11(S01234)	11(S01234)	11(S01234)	9(S0123)	IDC
**7**	-3(S05)	-3(S05)	-3(S05)	-3(S05)	-3(S05)	FA
**8**	9(S0123)	9(S0123)	9(S0123)	9(S0123)	9(S0123)	IDC
**9**	11(S01234)	11(S01234)	11(S01234)	11(S01234)	11(S01234)	IDC
**10**	11(S01234)	9(S0123)	9(S0123)	11(S01234)	9(S0123)	IDC
**11**	11(S01234)	11(S01234)	9(S0123)	9(S0123)	9(S0123)	IDC
**12**	9(S0123)	9(S0123)	9(S0123)	9(S0123)	9(S0123)	IDC
**13**	9(S0123)	9(S0123)	9(S0123)	9(S0123)	9(S0123)	IDC
**14**	-3(S05)	-3(S05)	-3(S05)	-3(S05)	-3(S05)	FA
**15**	-3(S05)	-3(S05)	-3(S05)	-3(S05)	-3(S05)	FA
**16**	-3(S05)	-3(S05)	-3(S05)	-3(S05)	-3(S05)	FA
**17**	9(S0123)	9(S0123)	9(S0123)	11(S01234)	9(S0123)	IDC
**18**	-3(S05)	-3(S05)	-3(S05)	-3(S05)	-3(S05)	FA
**19**	-3(S05)	-3(S05)	-3(S05)	-3(S05)	-3(S05)	FA
**20**	11(S01234)	9(S0123)	11(S01234)	11(S01234)	9(S0123)	IDC
**21**	9(S0123)	9(S0123)	9(S0123)	9(S0123)	9(S0123)	IDC
**22**	-3(S05)	0(S035)	-3(S05)	-3(S05)	0(S035)	FA

*IDC-invasive ductal carcinoma; FA-fibroadenoma.

## Discussion

There is an abundance of literature on PCI, especially pertaining to its use for breast screening. Many of these reports have yielded interesting results [36–44]. To our knowledge, however, the investigators in previous studies have not evaluated diagnostic signs in slices generated through PCI-CT reconstruction and prospectively summarised criteria similar to those established by BI-RADS for use in clinical diagnosis [29–31]. Therefore, our study emphasised the assessment of the diagnostic contribution of PCI-CT and summarises the characteristics of images for future prediction applications.

In our study, we analysed the diagnostic signs present in slices images with synchrotron radiation for the first time. We preliminarily proposed a diagnostic criterion that was evaluated by radiologists. All of the radiologists rendered the correct diagnosis. The total coincidence rate was 100%. We established 6 types of signs, ranging from sign S0 to S5, which were mainly characterised according to the distribution of adipose tissue and the edge characteristics of the tumour. S0 refers to scattered or lump-like adipose tissue around the tumour, which can occur in healthy or hyperplastic breasts. S1, termed the silhouette sign, refers to irregular margins or spiculation of the tumour. All fourteen malignant specimens exhibited S1. In contrast, benign tumours contain round or oval margins, termed a benign silhouette sign. All eight benign specimens exhibited a benign silhouette sign. S2 refers to scattered adipose tissue within the neoplastic area. All fourteen malignant specimens exhibited S2. Only two of the eight benign specimens presented with S2. The principal reason for this difference is the mode of neoplastic growth. The growth patterns of tumours include expansive growth, exogenous growth, and infiltrating growth. Malignant tumours present with infiltrating growth or exogenous growth. Benign tumours present with expansive growth or exogenous growth. A malignant tumour can infiltrate, invade, and destroy the adjacent non-neoplastic tissues. At the same time, some healthy adipose tissue can be encapsulated. Consequently, within a malignant tumour, there may be some scattered adipose tissue within the neoplastic area. In lobulated benign tumour, some adipose tissue may exist between various lobes. The morphology of this condition is completely different from that observed in malignant tumours, and it commonly presents with a line- or stripe-like shape, rather than a scattered shape. S3 refers to peripheral distorted and disordered adipose tissue. Malignancies that have infiltrated, invaded, and destroyed the adjacent tissues account for this diagnostic sign. S4, termed the finger pressure sign, refers to finger-like impressions in the interface region between the tumour and the surrounding structures. This feature is also caused by neoplastic infiltrating growth. Masses in an infiltrating growth mode grow into relatively healthy peripheral tissue and form several different sized nodules. S5, termed the border sign, refers to a natural outlining or demarcation of the tumour contour. This feature can only be observed in the majority of benign tumours. Most benign tumours developed within an enclosing fibrous capsule that separates the tumour tissue from the host tissue. The border sign can at least partially delineate a well-circumscribed shape of a benign tumour. S1-S4 are relatively specific to malignant tumours, while S1-S3 and S5 are relatively specific to benign tumours.

The future introduction of PCI-CT with synchrotron radiation into clinical practice may be difficult because of the limited field of view (FOV), temporal resolution, and spatial resolution. However, under limited conditions, our study has yielded explicit and significant results. PCI-CT has four main advantages. First, this technique yields improved soft-tissue contrast. Second, PCI-CT can partly reveal the fine structure of a breast tumour. Third, malignant and benign lesions such as IDCs and fibroadenomas, respectively, can be accurately differentiated. Finally, the method could improve early-stage breast cancer diagnosis.

Our study also had four main limitations. First, PC-CT has not revealed more detailed structures (e.g., fibrous connective tissue, necrosis, calcifications). This result may be caused by the limited resolution of the CCD. Moreover, to save experimental time and facilitate future practice, we purposely did not pursue higher spatial resolutions. But our summarised signs are adequate for clinical diagnosis. Second, artefacts may have influenced the image assessment. Slices from the reconstruction of original projections contain motion artefacts that were caused by specimen shrinkage during the experiment. Third, the number of specimens was limited. Given a larger sample size, more details may have been observed in the slices.
